# The Influence of Self-Regulation on Learner’s Behavioral Intention to Reuse E-Learning Systems: A Moderated Mediation Model

**DOI:** 10.3389/fpsyg.2021.763889

**Published:** 2021-10-18

**Authors:** Jia Xu, Xiaoyan Qiu

**Affiliations:** ^1^School of Political Science and Public Administration, Wuhan University, Wuhan, China; ^2^Institute of Teacher Education, Hunan University of Science and Engineering, Yongzhou, China

**Keywords:** self-regulation, behavioral intention to reuse, study engagement, peer collaboration, conservation of resource theory

## Abstract

Learners’ behavioral intention to reuse e-learning is of great significance to the implementation and management of e-learning in higher education. This study examined the relationship between self-regulation and behavioral intention to reuse by focusing on the mediating role of study engagement and the moderating role of peer collaboration. Based on a sample of 379 undergraduates from central China, we found that self-regulation positively influences behavioral intention to reuse *via* study engagement. In addition, moderated path analysis indicated that peer collaboration strengthened the direct effect of self-regulation on study engagement and its indirect effect on behavioral intention to reuse. Theoretical and practical implications of these findings are discussed as well.

## Introduction

Electronic learning (or e-learning) has gained increased attention during the COVID-19 lockdown. It has facilitated student learning and well-being by providing Internet access to teaching and learning programs (Radha et al., 2020) and psychological assistance (Zhao et al., 2020) in universities and schools. However, in many developing countries, e-learning is currently underused and still in its infant phase. A lack of use is resulting in resource wastage and the failure of e-learning systems generally (Almaiah et al., 2020). Therefore, improving learners’ behavioral intention to reuse e-learning systems is a major challenge for many universities (Alqahtani and Rajkhan, 2020; Radha et al., 2020).

During the past decades, scholars have identified several factors affecting learners’ adoption of the e-learning systems: performance expectancy, effort expectancy, social influence, and facilitating conditions (Zhang et al., 2020). Indeed, learning environment and learning expectations are associated with higher behavioral intention to reuse. However, e-learning might require a set of different strategies and skills to attain learning resources. Therefore, we focus on one specific learning strategy, namely, self-regulation. Self-regulated learning refers to a series of active and volitional behaviors that individual uses to accomplish a learning goal (Zimmerman, 1986; Panadero, 2017). Several studies have indicated that self-regulation can help the learner to control, manage, and plan their e-learning (Panadero, 2017; van Alten et al., 2020a). Nevertheless, the link between self-regulation and behavioral intention to reuse has rarely been investigated. Thus, it is crucial to explore the impacts of self-regulation on behavioral intention to reuse in the online environment.

The present study aimed to examine the effect of self-regulation on learners’ behavioral intention to reuse using the conservation of resource theory (Hobfoll, 1989). We proposed that when learners have the self-generated ability to control, manage, and plan the learning process, they show a greater behavioral intention to reuse e-learning systems. Moreover, we posited that self-regulation may be positively related to behavioral intention to reuse *via* study engagement. Wolters and Taylor (2012) suggested that there is a positive association between self-regulation and study engagement. Faisal et al. (2020) corroborated a positive relationship between cognitive and affective engagement and continued intention to use. Therefore, the present study chose study engagement as a mediator to understand how self-regulation affects positively the behavioral intention of learners to reuse.

The relationship between self-regulation, study engagement, and behavioral intention to reuse was also tested. Previous research has suggested that, when exploring the efficiency of e-learning, collaboration should also be considered (Papanikolaou and Boubouka, 2010; Cheng et al., 2011). Peer collaboration is defined as online support by group members to reach a common learning goal (Cheng et al., 2011). It has been uniformly regarded as a valuable resource that contributes to desired outcomes and should therefore be studied in most e-learning settings (Papanikolaou and Boubouka, 2010). It may contribute to learners’ motivation toward reusing e-learning systems, and so, its moderating role concerning the effects of self-regulation on behavioral intention to reuse was examined.

The present study complements the e-learning literature in several ways. First, it fills a critical research gap in the research on the relationship between self-regulation and behavioral intention to reuse. Previous studies of behavioral intention to reuse focused primarily on the institutional environment beyond individual factors (Cheng et al., 2011). Exploring the relationship between self-regulation and behavioral intention to reuse will help us have a more comprehensive understanding in terms of behavioral intention to reuse as self-regulation is an important indicator of effective learning and relates to outcomes such as academic motivation and self-efficacy (Lavasani et al., 2011).

Second, the present study contributes to the literature by showing why self-regulation affected behavioral intention to reuse. Although researchers have identified potential mediators, the motivational aspect (e.g., study engagement) of the influence of self-regulation has been neglected. Study engagement is worthy of investigation because motivation shapes learners’ reuse intention, and it is critical that educators understand how to enhance learners’ behavioral intention to reuse e-learning by encouraging them more. The other is that in the COVID-19 context, universities and schools should now always require that students give their full attention and energy to their e-learning tasks. Study engagement could be a possible mediator that transmits the influence of self-regulation on learners’ behavioral intention to reuse e-learning.

**Figure 1:**
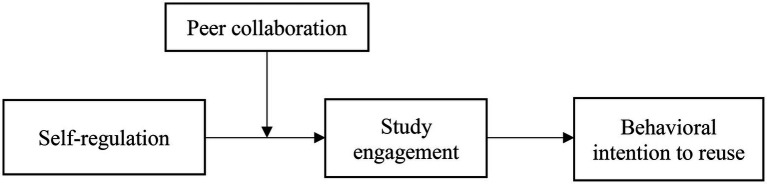
The proposed model.

Third, we identified an important environment resource (i.e., peer collaboration) as a key boundary condition in enhancing the positive effect of self-regulation on study engagement and behavioral intention to reuse. Previous studies have demonstrated the positive effects of self-regulation but not of potential boundary conditions (e.g., Yeh et al., 2019). Scholars should pay attention to how they might enhance the positive effects of self-regulation in e-learning. Our study wants to make a further step to identify a context factor in accelerating the positive effect of self-regulation. In particular, it introduced a moderated mediation model (Figure 1) positing study engagement as a mediator of the self-regulation’s effects on behavioral intention to reuse, with peer collaboration as a moderator of such effects.

## Theoretical Background and Hypotheses

### Conservation of Resource Theory

As has been noted, the present study builds on the conservation of resource theory (Hobfoll, 1989) to understand the link between self-regulation and behavioral intention to reuse. Conservation of resource theory assumes that individuals tend to protect and retain the resources they value. The resource could be anything that people think is valuable (Hobfoll, 1989). It suggests that when individuals lack resources, they tend to withdraw behavioral intention to minimize their loss. By contrast, individuals are interested in investing in more resources when such an investment generates returns. Because behavioral intention to reuse is considered a type of behavioral attitude toward acceptance and satisfaction (Chiu et al., 2005), which reflects a subjective expectation of how many resources learners can obtain by using e-learning systems, we hypothesized that self-regulation is positively related to behavioral intention to reuse.

### Self-Regulation and Behavioral Intention to Reuse

Behavioral intention refers to the degree to which an individual intends to perform some specific future behavior (Davis and Warshaw, 1992). In the context of e-learning, behavioral intention to reuse has been defined as the likelihood that learners will use an e-learning system again when it is made available to them (Budu et al., 2018). As learners’ willingness to adopt an e-learning system determines the success of any e-learning system (Li et al., 2012; Almaiah, 2018), understanding the factors that influence behavioral intention to reuse has been the focus of numerous e-learning studies (Al-Emran and Teo, 2020; Zhang et al., 2020). Previous research has identified several variables influencing learners’ decisions to use e-learning systems. These include perceived usefulness and perceived ease of use (Liaw, 2008); self-efficacy and attitudes toward use (Amin et al., 2015); the experience of e-learning (Mailizar et al., 2021); and social influence (Liaw, 2008).

Self-regulation is an important factor in effective learning and is widely used to explain learning outcomes in e-learning research. Self-regulated behaviors include but are not limited to environment structuring, goal setting, time management, help-seeking, task strategies, and self-evaluation (Barnard et al., 2009). According to the conservation of resource theory, the more learners perceive e-learning systems to be beneficial, the more they will use them. Because self-regulation enables learners to identify e-learning resources to achieve their goals (Wong et al., 2019), it can be assumed that it will increase their desire and belief to reuse them. Broadbent and Poon (2015) carried out a comprehensive analysis of self-regulation and concluded that self-regulation can help learners to achieve academic success in higher education settings. Various self-regulation outcomes have also been examined in previous studies (Panadero, 2017; van Alten et al., 2020a), for instance, satisfaction (van Alten et al., 2020b), learning performance (Zheng and Zhang, 2020), and academic achievement (Broadbent and Poon, 2015). Based on the above literature, we proposed the following hypothesis:

*Hypothesis* 1: Self-regulation is positively related to students’ behavioral intention to reuse e-learning systems.

### The Mediating Role of Study Engagement

The concept of study engagement has received increased attention in the e-learning literature (Rodgers, 2008; Moubayed et al., 2020). Study engagement is a psychological state of vigor, absorption, and dedication toward learning tasks (Lamborn et al., 1992) and reflects “the amount of effort dedicated to educational activities that bring out ideal performance” (Hu and Kuh, 2002, p. 555). Individual resources are an essential component of an individual’s engagement in their studies (Ryan, 2005). Previous literature has identified several resources that facilitate student engagement, such as interest in learning, belongingness (Hu and Kuh, 2002), deep learning, and relationship with others (Gunuc and Kuzu, 2015). Also, study engagement is associated with achievement in the academy and psychological well-being (Deakin Crick and Goldspink, 2014). Engagement in this study acts as a mediator that links self-regulation to behavioral intention to reuse. More explicitly, learners who can apply self-regulation strategies and skills will perceive a more positive state of passion and energy, which, in turn, leads to continuous use of e-learning systems.

The conservation of resource theory is a renowned paradigm for understanding study engagement (Gunuc and Kuzu, 2015). According to this theory (Hobfoll, 1989), engagement consumes energy, so individuals need to replenish their resources to maintain a high level (Xu et al., 2019a,b). The e-learning literature has also suggested that the e-learning environment is characterized by self-control, autonomy, and independence (Lynch and Dembo, 2004; Chiu et al., 2005); engagement in learning heavily relies on a student’s ability to control, manage, and plan learning programs (Ally, 2004). We assumed that self-regulation may help learners actively engage in their studies by adding their resources. First, self-regulation strategies could help learners acquire cognitive resources. For example, rehearsal and metacognitive strategies have been shown to be positively related to the acquisition and retention of knowledge (Yukselturk and Bulut, 2007). Second, self-regulation enables learners to manage their emotions in the pursuit of studying goals, which provides emotional resources for student engagement. Studies have shown that strategies of regulating emotions, such as situation selection, situation modification, attentional deployment, and response modulation, are linked to learners’ positive emotions (Webster and Hadwin, 2015). Therefore, it can be seen that self-regulation may facilitate student engagement.

Furthermore, study engagement can affect students’ behavioral intention to reuse e-learning systems. Engagement is characterized by dedication, absorption, and vigor. Dedication reflects individuals’ desire to invest effort in studying. Absorption refers to the state individuals enter when they are completely immersed in a task. Vigor emphasizes one’s excitement and interest in study (Schaufeli et al., 2002a; Axelson and Flick, 2010). Study engagement involves a series of activities, including behavioral engagement (e.g., concentration), emotional engagement (e.g., belonging and excitement), and cognitive engagement (e.g., mental effort; Webster and Hadwin, 2015). In terms of the conservation of resource theory, learners tend to reuse e-learning systems when they perceive them to have value. Close engagement will make learners more persistent in their e-learning. Previous research has suggested that study engagement leads to a higher return in terms of satisfaction (Webster and Hadwin, 2015); academic performance (Carini et al., 2006); student success; and retention (Wyatt, 2011). In light of the above, we proposed the following hypotheses:

*Hypothesis* 2: Study engagement mediates the relationship between self-regulation and behavioral intention to reuse e-learning systems.

### The Moderating Role of Peer Collaboration

Although we expected that self-regulation would positively influence study engagement, which in turn impacts behavioral intention to reuse, the conservation of resource theory (Hobfoll, 1989) indicates that environmental factors influence how learners manage and make use of self-regulation strategies and skills and obtain new resources. In e-learning settings, peer collaboration is regarded as a form of learner support where students are encouraged to exchange ideas with other students (Biasutti, 2011). It is also an important influence on mental health (Kim et al., 2020). Drawing on these insights, we proposed that peer collaboration presents an environmental resource that may strengthen the positive impact of self-regulation on study engagement.

Learners with high levels of peer collaboration tend to possess greater learning resources and are subsequently more capable of engaging with their studies. Scholars have suggested that peer collaboration facilitates both academic and social integration (Papanikolaou and Boubouka, 2010; Al-Abri et al., 2017). Learners with high levels of peer collaboration are more likely than learners with low levels of peer collaboration to perceive their self-regulation strategies and skills as an effective way to acquire more resources. Logically, learners who employ self-regulation already have the effective learning ability to obtain resources with which to invest in study engagement. If learners also receive a high level of peer support, they will become more confident in participating in e-learning activities. Peer collaboration not only helps learners transfer information and ideas to improve their self-regulation but also helps them to cooperate, improve learning climates, and enhance learning satisfaction. As a result, the relationship between self-regulation and study engagement should be stronger for learners with high levels of peer collaboration. By contrast, learners with low levels of peer collaboration should be more vulnerable to insufficient learning-related resources and less capable of investing their resources to enhance the outcomes of self-regulation, thus decreasing the effectiveness of self-regulation for integrating personal and contextual resources. Accordingly, we proposed the following hypothesis:

*Hypothesis* 3: Peer collaboration moderates the relationship between self-regulation and study engagement; this relationship is more positive when peer collaboration is high rather than low.

Given that peer collaboration moderates the relation between self-regulation and study engagement and considering that study engagement is positively associated with behavioral intention to reuse, it is logical to suggest that peer collaboration also moderates the strength of the mediating mechanism for study engagement in the relationship between self-regulation and behavioral intention to reuse, which presents a moderated mediation model (Edwards and Lambert, 2007). As previously mentioned, a stronger relation between self-regulation and behavioral intention to reuse will appear among learners with high levels of peer collaboration. Hence, the indirect effect of self-regulation on behavioral intention to reuse *via* study engagement may be stronger for a learner who collaborates with their peers. In particular, when a student has a high level of peer collaboration, the indirect effect of self-regulation on behavioral intention to reuse should be stronger. However, when a student is lacking such a high level of collaboration, self-regulation is less influential in promoting study engagement; consequently, the indirect effect of self-regulation on behavioral intention to reuse should be weaker. In light of the above, we proposed the following hypothesis:

*Hypothesis* 4: Peer collaboration moderates the mediating effect of study engagement on the relationship between self-regulation and behavioral intention to reuse such that the indirect effect of self-regulation on behavioral intention to reuse *via* study engagement is stronger for learners with a high level of peer collaboration than for learners with a low level of peer collaboration.

## Materials and Methods

### Respondents and Procedures

We drew our sample from one large university in central China. The e-learning programs were part of the students’ regular school curriculum. The questionnaires were distributed among undergraduates within the e-learning system. They were asked to complete a self-assessment of their self-regulation, study engagement, peer collaboration, and behavioral intention to reuse the e-learning system. All participants were assured that all data would be anonymous. They signed an electronic consent form agreeing to their participation in the survey.

With the help of their professors, 400 students who engaged in e-learning activities were invited to take part. From this sample, 379 valid samples were retained a response rate of 94.75%. The average age was 20.72years (*SD*=2.17); 67.81% were male, and 32.19% were female. In terms of grades, 19.90% were freshmen, 33.25% were sophomores, 24.01% were juniors, 14.77% were seniors, and 8.97% were students in the fifth year. Their majors were as follows: 8.18%, humanities; 33.25%, economics and management; 15.57%, science; 54.35%, engineering; 1.85%, law; 3.96%, medicine; and 3.69%, education.

### Measurement

#### Self-Regulation

Self-regulation was measured, using a 24-item scale from Barnard et al. (2009). Example items include the following: goal setting – “I set standards for my assignments in online courses”; environment structuring – “I know where I can study most efficiently for online courses”; task strategies – “I work extra problems in my online courses in addition to the assigned ones to master the course content”; time management – “I allocate extra studying time for my online courses because I know it is time-demanding”; help-seeking – “I find someone who is knowledgeable in course content so that I can consult with him or her when I need help”; and self-evaluation – “I summarize my learning in online courses to examine my understanding of what I have learned.” Participants were asked to respond on a seven-point scale ranging from 1 (*strongly disagree*) to 7 (*strongly agree*) at Time 1. The internal consistency is *α*=0.96.

#### Study Engagement

We used a 17-item scale developed by Schaufeli et al. (2002b) to measure the study engagement of learners. It has three dimensions, including vigor, dedication, and absorption. Example items include the following: vigor – “When I study, I feel like I am bursting with energy”; dedication – “I am enthusiastic about my studies”; and absorption – “I feel happy when I am studying intensively.” Participants were asked to respond on a seven-point scale ranging from 1 (*strongly disagree*) to 7 (*strongly agree*). The internal consistency of the study engagement scale is *α*=0.97.

#### Behavioral Intention to Reuse

We measured behavioral intention to reuse with Li et al. (2012) three-item scale. Example items include “Assuming that I had access to the e-learning system, I intend to reuse it.” Participants were asked to respond on a seven-point scale ranging from 1 (*strongly disagree*) to 7 (*strongly agree*). The internal consistency of the scale is *α*=0.89.

#### Peer Collaboration

Peer collaboration was measure through a five-item scale developed by Zhang et al. (2014). An example item is “My group members and I actively work together to help each other understand the learning task.” Participants were asked to respond on a seven-point scale ranging from 1 (*strongly disagree*) to 7 (*strongly agree*). The internal consistency of the scale is *α*=0.94.

#### Control Variables

Socio-demographics were considered these variables as potential control variables. Demographic variables included age, gender (1=*male*, 2=*female*), grade (1=*first year*, 2=*second year*, 3=*third year*, 4=*fourth year*, 5=*fifth year*), and major (1=*humanity*, 2=*economy and management*, 3=*science*, 4=*engineer*, 5=*law*, 6=*medicine*, 7=*education*).

## Results

### Preliminary Analyses

First, the confirmatory factor analysis with Mplus7.0 was used to examine the distinctiveness of the latent variables. The results showed that a theorized four-factor model distinguishing between self-regulation, study engagement, behavioral intention to reuse, and peer collaboration was a better fit to the data (*χ*^2^=482.34, *df*=113, *p*<0.001; *CFI*=0.94; *TLI*=0.93; *RMSEA*=0.09; *SRMR*=0.04) than alternative models: (a) a four-factor model in which self-regulation and study engagement were combined into one factor (*χ*^2^=1211.41, *df*=116, *p*<0.001; *CFI*=0.83; *TLI*=0.81; *RMSEA*=0.16; *SRMR*=0.07); (b) a three-factor model in which self-regulation and behavioral intention to reuse were combined into one factor (*χ*^2^=973.54, *df*=116, *p*<0.001; *CFI*=0.87; *TLI*=0.85; *RMSEA*=0.14; *SRMR*=0.07); (c) a two-factor model in which self-regulation, study engagement, and behavioral intention to reuse were combined into one factor (*χ*^2^=1542.61, *df*=118, *p*<0.001; *CFI*=0.78; *TLI*=0.75; *RMSEA*=0.18; *SRMR*=0.08); and (d) a one-factor model in which all latent variables were combined into one factor (*χ*^2^=2152.89, *df*=119, *p*<0.001; *CFI*=0.69; *TLI*=0.65; *RMSEA*=0.21; *SRMR*=0.09). The results illustrated that the participants were able to distinguish the studied variables.

Finally, we used the unmeasured latent method factor to examine the presence of common method variance (CMV; Podsakoff et al., 2003). Compared with the theorized four-factor model (*χ*^2^=482.34, *df*=113, *p*<0.001; *CFI*=0.94; *TLI*=0.93; *RMSEA*=0.09; *SRMR*=0.04), the indices of the five-factor model with all items loading on a latent common method (*χ*^2^=320.04, *df*=97, *p*<0.001; *CFI*=0.96; *TLI*=0.95; *RMSEA*=0.08; *SRMR*=0.03) were not significantly improved. The findings indicated that the data were not inflated with CMV.

### Descriptive Statistics and Correlations

The means, SDs, and correlations of the variables are provided in Table 1.

**Table 1:** Descriptive statistics and inter-correlations among variable.

	*M*	*SD*	1	2	3	4
1. Self-regulation	5.05	1.15	NA			
2. Peer collaboration	4.99	1.30	0.73^***^	NA		
3. Study engagement	4.81	1.30	0.72^***^	0.67^***^	NA	
4. Behavioral intentions to reuse	5.42	1.20	0.56^***^	0.56^***^	0.67^***^	NA

*N*=379. NA, not applicable.

*** *p*<0.001.

### Hypothesis Testing

**Figure 2:**
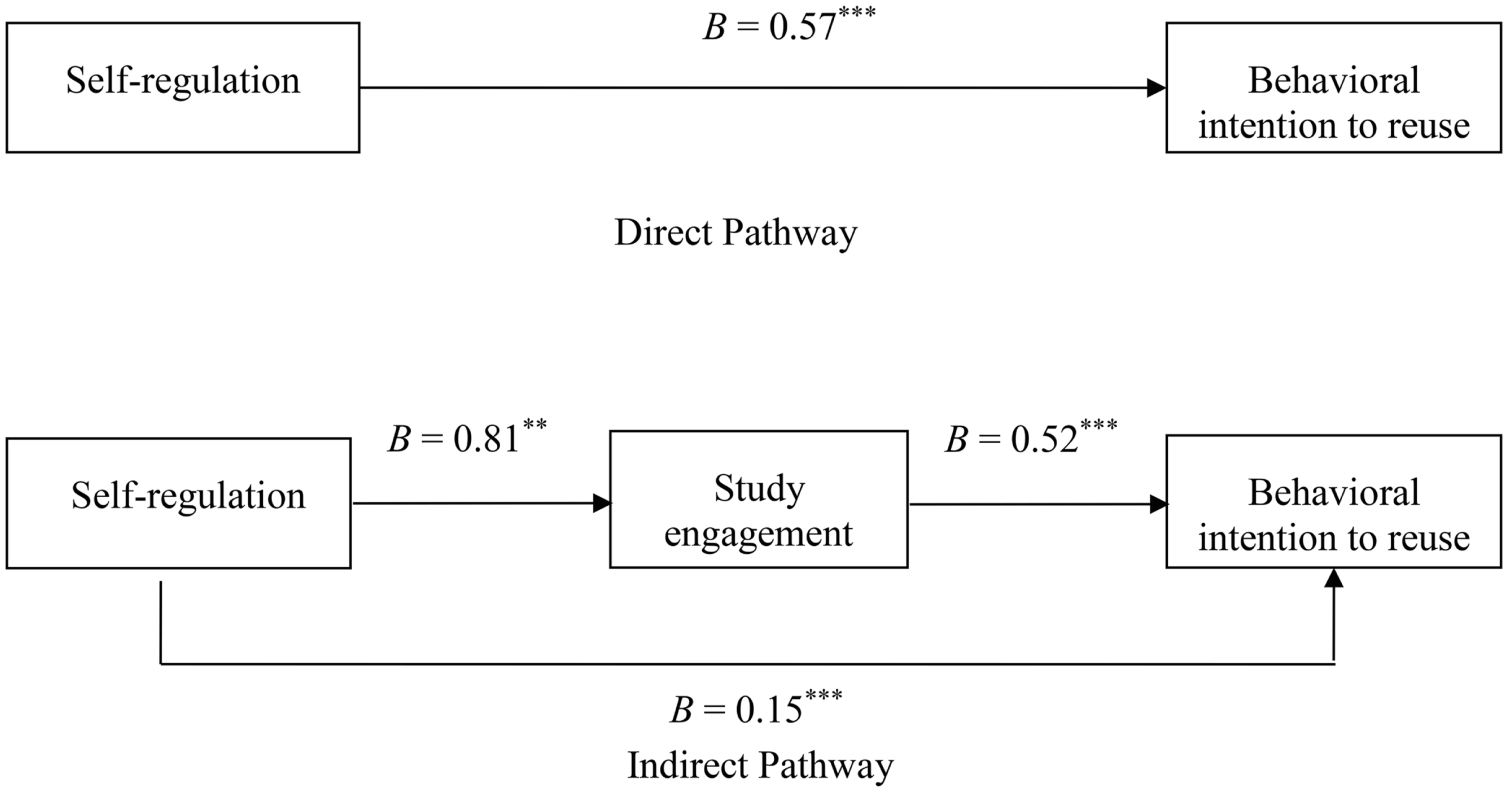
The mediating role of study engagement. Unstandardized path coefficients were reported; ^***^p<0.001 and ^**^p<0.01.

Hypothesis 1 stated that self-regulation was positively associated with behavioral intention to reuse e-learning. Hypothesis 2 further argued that study engagement mediated the relationship. The hierarchical regression analysis run by the Statistical Package for the Social Sciences (SPSS) was used to test Hypothesis 1. The mediation procedure macro developed by Preacher and Hayes (2008) was used to test Hypothesis 2. The results of hypothesis tests are summarized in Figure 2.

The direct pathway in Figure 2 shows that self-regulation was positively associated with behavioral intention to reuse (*B*=0.57, *SE*=0.05, *p*<0.001), controlling for socio-demographic variables. Thus, Hypothesis 1 was supported. The indirect pathway in Figure 2 further shows that self-regulation was positively related to study engagement (*B*=0.81, *SE*=0.04, *p*<0.001), which in turn was positively associated with behavioral intention to reuse (*B*=0.52, *SE*=0.05, *p*<0.001). The 95% CI for the indirect effect was [0.32, 0.52], with an average of 0.42, and did not include 0. Thus, Hypothesis 2 was supported. Additionally, Figure 2 shows that the direct effect of self-regulation on behavioral intention to reuse was still significant (*B*=0.15, *SE*=0.06, *p*<0.001). According to Baron and Kenny (1986), study engagement partially mediated the positive relationship between self-regulation and behavioral intention to reuse.

Hypothesis 3 stated that peer collaboration moderated the effect of self-regulation on study engagement. Hypothesis 4 further stated that peer collaboration moderated the indirect effect of self-regulation on behavioral intention to reuse *via* study engagement. The moderation and moderated mediation procedure macro developed by Preacher and Hayes (2008) was used to test Hypothesis 3–4. The results of hypothesis tests are summarized in Table 2.

**Table 2:** The results of examining Hypothesis 3 and 4.

Model	Conditional effect	95% LL	95% UL
Moderated model (H3)			
Low (−1sd)	0.44^***^ (0.06)	0.31	0.56
High (+1sd)	0.63^***^ (0.06)	0.51	0.75
Moderated mediation model (H4)			
Low (−1sd)	0.23^***^ (0.05)	0.15	0.32
High (+1sd)	0.33^***^ (0.05)	0.24	0.42

Demographical variables are controlled; Bootstrap sample size=5,000; the numbers in the parentheses are standard errors; +1sd=1.30 and −1sd=−1.30 for centered peer collaboration; tests were based on bias-corrected confidence intervals.

*** *p*<0.001.

**Figure 3:**
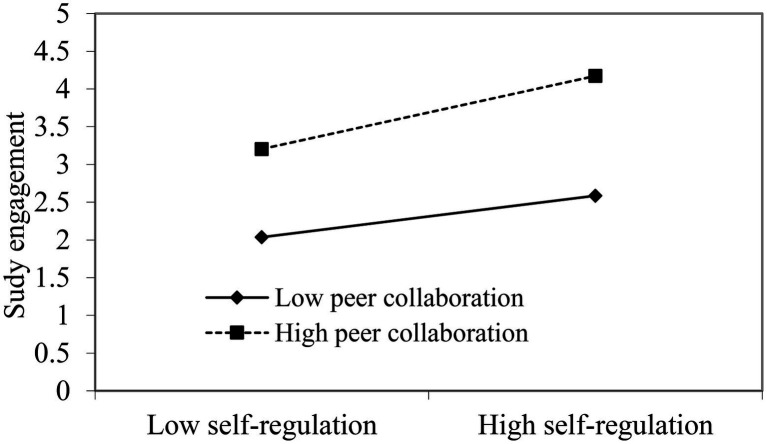
The moderating role of peer collaboration on the relationship between self-regulation and study engagement.

The moderation analysis shows that the interaction of self-regulation and peer collaboration positively predicted study engagement (*B*=0.08, *SE*=0.02, *p*<0.01) when the controls are controlled. Table 2 shows that the simple slope test showed that simple slope was 0.44 (*SE*=0.06, *p*<0.001) at low level of peer collaboration (−1sd=−1.30), and it was 0.63 (*SE*=0.06, *p*<0.001) at high level of peer collaboration (+1sd=1.30). Figure 3 clearly illustrates the moderating role of peer collaboration in the self-regulation-study engagement association. Thus, Hypothesis 3 was supported.

**Figure 4:**
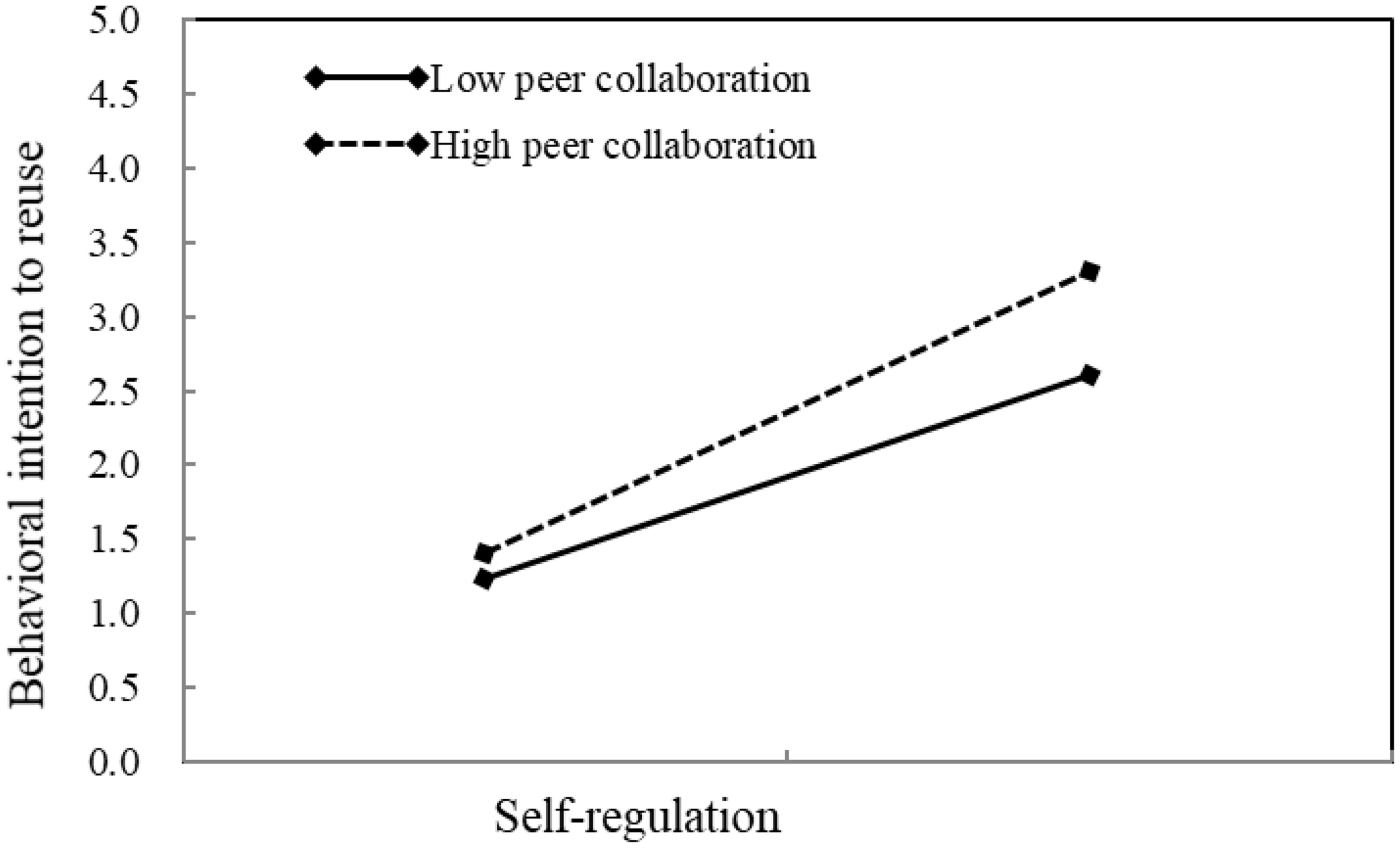
The moderating role of peer collaboration on the indirect effect.

Table 2 also shows that the indirect effects of self-regulation on behavioral intention to reuse *via* study engagement varied significantly across different levels of peer collaboration. The indirect effect of self-regulation on behavioral intention to reuse was more significant when peer collaboration was high (*B*=0.33, *SE*=0.05, *p*<0.001) than those when peer collaboration was low (*B*=0.23, *SE*=0.05, *p*<0.001). Figure 4 clearly illustrates the moderating role of peer collaboration in the indirect effect of self-regulation on behavioral intention to reuse *via* study engagement. Hypothesis 4 was therefore supported.

## Discussion

All the hypotheses proposed in this research have been verified, and the results include the following points. First, self-regulation influenced students’ behavioral intention to reuse. Second, study engagement plays a mediating role between self-regulation and behavioral intention to reuse. Third, peer collaboration moderates the positive effect of self-regulation on study engagement and behavioral intention to reuse. Specifically, learners with high peer collaboration will perceive higher study engagement and thus enhance behavioral intention to reuse.

### Theoretical Implications

First, our research contributes to the e-learning literature by proposing and confirming the effect of self-regulation on behavioral intention to reuse an e-learning system. E-learning research has highlighted the importance of considering self-regulation strategies and skills because they influence how learners manage, plan, and reflect learning process (Jansen et al., 2019, 2020). These studies appear particularly valuable for identifying ways to improve the success of e-learning. However, in many cases, learners’ continuance usage of e-learning, such as behavioral intention to reuse, are of limited study for the outcomes of self-regulation. The current study, enriching the effects of self-regulation, indicates that self-regulation would influence behavioral intention to reuse e-learning. This is one of the limited studies that establish the relationship between self-regulation and behavioral intention to reuse.

Second, our study further contributes to the research on e-learning by identifying study engagement as a mediator linking self-regulation and behavioral intention to reuse. Given the importance of behavioral intention to reuse for e-learning success, previous studies have mostly focused on several important influencing factors (e.g., performance expectancy, effort expectancy, and social influence; Zhang et al., 2020), and what has been missing from research is study engagement. This construct represents learners’ passion and dedication to spend their time and energy on their study work. Consequently, a better understanding of the role of study engagement in influencing behavioral intention to reuse is needed. Drawing on conservation of resource theory (Hobfoll, 1989), we find that learners who can employ self-regulation strategies tend to experience emotional, cognitive, and physical engagement, and hence, it is more likely for them to reuse e-learning.

Third, by applying a moderated mediation framework, we revealed the moderating role of peer collaboration, supporting the applicability of the conservation of resource perspective in explaining the link between self-regulation and behavioral intention to reuse through study engagement. This study extends the self-regulation literature by answering the call to examine boundary conditions for the effectiveness of self-regulation. Self-regulation has been established as a strong predictor of positive outcomes of e-learning (El-Adl and Alkharusi, 2020; Jivet et al., 2020). Yet, very little research has focused on the potential boundary conditions that either promote or hinder the positive effects of self-regulation. This study suggests that high peer collaboration strengthens the positive effect of self-regulation on behavioral intention to reuse and study engagement. Learners who learn in an environment that provides peer collaboration have highly positive learning support. Thus, we can view high peer collaboration as part of a positive external environment. Our findings provide insights into how peer collaboration, as a contextual factor to facilitate self-regulation, can boost the increased intention to adopt e-learning systems.

### Practical Implications

First, for educators, more attention should be paid to self-regulation. Learners’ self-regulation has always been regarded as an essential goal perused by e-learning. Self-regulation undoubtedly increases the outcomes of e-learning; moreover, the results of this study showed that self-regulation has a positive effect on the behavioral intention to reuse of the learners. This indicates that if educators cultivate learners’ self-regulation strategies and skills, learners’ adoption of e-learning will be increased. Therefore, educators should focus on how to improve self-regulation ability among students when they want to increase their enthusiasm for reusing the e-learning system. Specifically, mental effort (van Gog et al., 2020) may be an effective way to help students to improve self-regulation in higher education.

Second, appropriate ways should be chosen to improve the study engagement of the learners. Activities can be held to increase the contextual resources of learners to make more resources, which is beneficial to the study engagement of learners. Universities are expected to increase students’ psychological influences, such as teachers and teaching practice, feeling part of a learning community, curriculum, and assessment, which greatly benefits the generation of study engagement (Kahu, 2013).

Finally, the different levels of peer collaboration of learners should be fully explored and the collaborative learning activities (Laal and Ghodsi, 2012; El Mhouti et al., 2017) and the social integration process (Blau, 1960; Severiens and Schmidt, 2009) should be taken. For example, more consideration should be given to social and psychological support (Li et al., 2018), such as the contacts between peers, cooperative work, a cooperative learning environment, and quality of teacher-student, which could increase environmental resources for learners.

## Limitation and Future Research

With all research, this study has several limitations. First, the data of this study were collected at the same time point, which may only reflect the current self-regulation, peer collaboration, study engagement, and behavioral intention to reuse. Although it reflects the relationship among the variables, it cannot fully reflect the causal relationship among self-regulation, study engagement, and behavioral intention to reuse. In addition, the data were collected in the metropolitan area of central China, which may limit the generalization of our findings to far-away places or other cultures. It is suggested future research test our results in other contexts. Third, we relied on undergraduates’ self-reports on all variables, which may raise concerns about CMV (Lindell and Whitney, 2001). We thus encourage future research to focus on teacher rating of behavioral intention to reuse to reduce CMV. Last, this study discusses the relationship between self-regulation and the behavioral intention to reuse of learners based on conservation of resource theory, without considering the role of the theory of social integration (Blau, 1960) in explaining this issue. Theories, such as self-determination theory (Ryan and Deci, 2000), also can help researchers explain the effect of self-regulation. So, future research can combine other theories to more fully reflect the influence of self-regulation and study engagement on behavioral intention to reuse.

## Data Availability Statement

The raw data supporting the conclusions of this article will be made available by the authors, without undue reservation.

## Ethics Statement

Ethical review and approval was not required for the study on human participants in accordance with the local legislation and institutional requirements. Written informed consent for participation was not required for this study in accordance with the national legislation and the institutional requirements.

## Author Contributions

JX developed the study design and drafted the manuscript. XQ collected the data and performed the statistical analysis. All authors contributed to the article and approved the submitted version.

## Funding

This project was supported by MOE (Ministry of Education in China) Project of Humanities and Social Sciences (grant no. 19YJC630190) and the Fundamental Research Funds for the Central Universities (grant no. 1203-413000068/2020AI010). Project of Excellent Online Open Courses of Hunan University of Science and Engineering, Grant No. 201920-13.

## Conflict of Interest

The authors declare that the research was conducted in the absence of any commercial or financial relationships that could be construed as a potential conflict of interest.

## Publisher’s Note

All claims expressed in this article are solely those of the authors and do not necessarily represent those of their affiliated organizations, or those of the publisher, the editors and the reviewers. Any product that may be evaluated in this article, or claim that may be made by its manufacturer, is not guaranteed or endorsed by the publisher.

## References

[ref1] Al-Abri A. Jamoussi Y. Kraiem N. Al-Khanjari Z. (2017). Comprehensive classification of collaboration approaches in E-learning. Telematics Inform. 34, 878–893. doi: 10.1016/j.tele.2016.08.006

[ref3] Al-Emran M. Teo T. (2020). Do knowledge acquisition and knowledge sharing really affect e-learning adoption? An empirical study. Educ. Inf. Technol. 25, 1983–1998. doi: 10.1007/s10639-019-10062-w

[ref2] Ally M. (2004). “Foundations of educational theory for online learning,” in Educational Practice Theory. Vol. 2. ed. Anderson T. (Edmonton, AB: Athabasca University Press), 15–44.

[ref67] Almaiah M. A. (2018). Acceptance and usage of a mobile information system services in University of Jordan. Educ. Inf. Technol. 23, 1873–1895. doi: 10.1007/s10639-018-9694-6

[ref4] Almaiah M. A. Al-Khasawneh A. Althunibat A. (2020). Exploring the critical challenges and factors influencing the E-learning system usage during COVID-19 pandemic. Educ. Inf. 25, 5261–5280. doi: 10.1007/s10639-020-10219-y, PMID: 32837229 PMC7243735

[ref5] Alqahtani A. Y. Rajkhan A. A. (2020). E-learning critical success factors during the covid-19 pandemic: a comprehensive analysis of e-learning managerial perspectives. Educ. Sci. 10:216. doi: 10.3390/educsci10090216

[ref6] Amin M. K. Akter A. Azhar A. Akter S. (2015). “Applying TAM to understand students’ behavioral Intention to use E-learning system: an empirical evidence from Bangladesh.” in *18th International Conference on Computer and Information Technology (ICCIT)*; October 25–26, 2016.

[ref7] Axelson R. D. Flick A. (2010). Defining student engagement. Change 43, 38–43. doi: 10.1080/00091383.2011.533096

[ref8] Barnard L. Lan W. Y. To Y. M. Paton V. O. Lai S. L. (2009). Measuring self-regulation in online and blended learning environments. Internet High. Educ. 12, 1–6. doi: 10.1016/j.iheduc.2008.10.005

[ref9] Baron R. M. Kenny D. A. (1986). The moderator–mediator variable distinction in social psychological research: conceptual, strategic, and statistical considerations. J. Pers. Soc. Psychol. 51:1173. doi: 10.1037/0022-3514.51.6.1173, PMID: 3806354

[ref10] Biasutti M. (2011). The student experience of a collaborative e-learning university module. Comput. Sci. Educ. 57, 1865–1875. doi: 10.1016/j.compedu.2011.04.006

[ref11] Blau P. M. (1960). A theory of social integration. Am. J. Sociol. 65, 545–556. doi: 10.1086/222785

[ref12] Broadbent J. Poon W. L. (2015). Self-regulated learning strategies & academic achievement in online higher education learning environments: a systematic review. Internet High. Educ. 27, 1–13. doi: 10.1016/j.iheduc.2015.04.007

[ref13] Budu K. W. A. Yinping M. Mireku K. K. (2018). Investigating the effect of behavioral intention on e-learning systems usage: empirical study on tertiary education institutions in Ghana. Mediterr. J. Soc. Sci. 9, 201–216. doi: 10.2478/mjss-2018-0062

[ref14] Carini R. M. Kuh G. D. Klein S. P. (2006). Student engagement and student learning: testing the linkages. Stud. High. Educ. 47, 1–32. doi: 10.1007/s11162-005-8150-9

[ref15] Cheng B. Wang M. Yang S. J. Peng J. (2011). Acceptance of competency-based workplace e-learning systems: effects of individual and peer learning support. Comput. Sci. Educ. 57, 1317–1333. doi: 10.1016/j.compedu.2011.01.018

[ref16] Chiu C. M. Hsu M. H. Sun S. Y. Lin T. C. Sun P. C. (2005). Usability, quality, value and e-learning continuance decisions. Comput. Sci. Educ. 45, 399–416. doi: 10.1016/j.compedu.2004.06.001

[ref17] Davis F. D. Warshaw P. R. (1992). What do intention scales measure? J. Gen. Psychol. 119, 391–407. doi: 10.1080/00221309.1992.9921181

[ref18] Deakin Crick R. Goldspink C. (2014). Learner dispositions, self-theories and student engagement. Br. J. Educ. Stud. 62, 19–35. doi: 10.1080/00071005.2014.904038

[ref19] Edwards J. R. Lambert L. S. (2007). Methods for integrating moderation and mediation: a general analytical framework using moderated path analysis. Psychol. Methods 12, 1–22. doi: 10.1037/1082-989X.12.1.1, PMID: 17402809

[ref20] El Mhouti A. Nasseh A. Erradi M. Vasquèz J. M. (2017). Enhancing collaborative learning in web 2.0-based e-learning systems: a design framework for building collaborative e-learning contents. Educ. Inf. 22, 2351–2364. doi: 10.1007/s10639-016-9545-2

[ref21] El-Adl A. Alkharusi H. (2020). Relationships between self-regulated learning strategies, learning motivation and mathematics achievement. Cypriot J. Educ. Sci. 15, 104–111. doi: 10.18844/cjes.v15i1.4461

[ref22] Faisal C. M. N. Fernandez-Lanvin D. De Andrés J. Gonzalez-Rodriguez M. (2020). Design quality in building behavioral intention through affective and cognitive involvement for e-learning on smartphones. Internet Res. 10:216. doi: 10.1108/INTR-05-2019-0217

[ref23] Gunuc S. Kuzu A. (2015). Student engagement scale: development, reliability and validity. Assess. Eval. High. Educ. 40, 587–610. doi: 10.1080/02602938.2014.938019

[ref24] Hobfoll S. E. (1989). Conservation of resources: a new attempt at conceptualizing stress. Am. Psychol. 44, 513–524. doi: 10.1037/0003-066X.44.3.513, PMID: 2648906

[ref25] Hu S. Kuh G. D. (2002). Being (dis) engaged in educationally purposeful activities: the influences of student and institutional characteristics. Stud. High. Educ. 43, 555–575. doi: 10.1023/A:1020114231387

[ref26] Jansen R. S. van Leeuwen A. Janssen J. Conijn R. Kester L. (2020). Supporting learners' self-regulated learning in massive open online courses. Comput. Sci. Educ. 146:103771. doi: 10.1016/j.compedu.2019.103771

[ref27] Jansen R. S. Van Leeuwen A. Janssen J. Jak S. Kester L. (2019). Self-regulated learning partially mediates the effect of self-regulated learning interventions on achievement in higher education: a meta-analysis. Educ. Res. Rev. 28:100292. doi: 10.1016/j.edurev.2019.100292

[ref28] Jivet I. Scheffel M. Schmitz M. Robbers S. Specht M. Drachsler H. (2020). From students with love: an empirical study on learner goals, self-regulated learning and sense-making of learning analytics in higher education. Internet High. Educ. 47:100758. doi: 10.1016/j.iheduc.2020.100758

[ref29] Kahu E. R. (2013). Framing student engagement in higher education. Stud. High. Educ. 38, 758–773. doi: 10.1080/03075079.2011.598505

[ref30] Kim S. Y. Lim K. M. Kim S. J. Kim G. U. Kim B. (2020). Embracing and growing as a peer support provider: an analysis of participants’ experience in a peer support program based on the recovery model of mental illness. Int. J. Ment. Health Promot. 22, 261–270. doi: 10.32604/IJMHP.2020.013279

[ref31] Laal M. Ghodsi S. M. (2012). Benefits of collaborative learning. Procedia Soc. Behav. Sci. 31, 486–490. doi: 10.1016/j.sbspro.2011.12.091

[ref32] Lamborn S. Newmann F. Wehlage G. (1992). “The significance and sources of student engagement,” in Student Engagement and Achievement in American Secondary Schools. ed. Newman F. M. (New York: Teachers College Press), 11–39.

[ref33] Lavasani M. G. Mirhosseini F. S. Hejazi E. Davoodi M. (2011). The effect of self-regulation learning strategies training on the academic motivation and self-efficacy. Procedia Soc. Behav. Sci. 29, 627–632. doi: 10.1016/j.sbspro.2011.11.285

[ref34] Li Y. Duan Y. Fu Z. Alford P. (2012). An empirical study on behavioural intention to reuse e-learning systems in rural China. Br. J. Educ. Technol. 43, 933–948. doi: 10.1111/j.1467-8535.2011.01261.x

[ref35] Li J. Han X. Wang W. Sun G. Cheng Z. (2018). How social support influences university students’ academic achievement and emotional exhaustion: the mediating role of self-esteem. Learn. Individ. Differ. 61, 120–126. doi: 10.1016/j.lindif.2017.11.016

[ref36] Liaw S.-S. (2008). Investigating students’ perceived satisfaction, behavioral intention, and effectiveness of e-learning: a case study of the blackboard system. Comput. Sci. Educ. 51, 864–873. doi: 10.1016/j.compedu.2007.09.005

[ref37] Lindell M. K. Whitney D. J. (2001). Accounting for common method variance in cross-sectional research designs. Int. J. Appl. Psychol. 86, 114–121. doi: 10.1037/0021-9010.86.1.114 11302223

[ref38] Lynch R. Dembo M. (2004). The relationship between self-regulation and online learning in a blended learning context. Int. Rev. Res. Open Dis. 5, 1–16. doi: 10.19173/irrodl.v5i2.189

[ref39] Mailizar M. Almanthari A. Maulina S. (2021). Examining teachers’ behavioral intention to use E-learning in teaching of mathematics: an extended TAM model. Contemp. Educ. Technol. 13:ep298. doi: 10.30935/cedtech/9709 PMC8079853 33935579

[ref40] Moubayed A. Injadat M. Shami A. Lutfiyya H. (2020). Student engagement level in an e-learning environment: clustering using k-means. Am. J. Dist. Educ. 34, 137–156. doi: 10.1080/08923647.2020.1696140

[ref41] Panadero E. (2017). A review of self-regulated learning: six models and four directions for research. Front. Psychol. 8:422. doi: 10.3389/fpsyg.2017.00422, PMID: 28503157 PMC5408091

[ref42] Papanikolaou K. Boubouka M. (2010). Promoting collaboration in a project-based e-learning context. J. Res. Technol. Educ. 43, 135–155. doi: 10.1080/15391523.2010.10782566

[ref43] Podsakoff P. M. MacKenzie S. B. Lee J. Y. Podsakoff N. P. (2003). Common method biases in behavioral research: a critical review of the literature and recommended remedies. Int. J. Appl. Psychol. 88, 879–903. doi: 10.1037/0021-9010.88.5.879 14516251

[ref44] Preacher K. J. Hayes A. F. (2008). Asymptotic and resampling strategies for assessing and comparing indirect effects in multiple mediator models. Behav. Res. Methods 40, 879–891. doi: 10.3758/BRM.40.3.879, PMID: 18697684

[ref45] Radha R. Mahalakshmi K. Kumar V. S. Saravanakumar A. (2020). E-learning during lockdown of Covid-19 pandemic: a global perspective. Int. J. Control Autom. 13, 1088–1099.

[ref46] Rodgers T. (2008). Student engagement in the e-learning process and the impact on their grades. Int. J. Cyber Soc. Educ. 1, 143–156.

[ref47] Ryan J. F. (2005). Institutional expenditures and student engagement: a role for financial resources in enhancing student learning and development? Stud. High. Educ. 46, 235–249. doi: 10.1007/s11162-004-1601-x

[ref48] Ryan R. M. Deci E. L. (2000). Self-determination theory and the facilitation of intrinsic motivation, social development, and well-being. Am. Psychol. 55, 68–78. doi: 10.1037/0003-066X.55.1.68, PMID: 11392867

[ref49] Schaufeli W. B. Martinez I. M. Pinto A. M. Salanova M. Bakker A. B. (2002a). Burnout and engagement in university students: a cross-national study. J. Cross-Cult. Psychol. 33, 464–481. doi: 10.1177/0022022102033005003

[ref50] Schaufeli W. B. Salanova M. González-Romá V. Bakker A. B. (2002b). The measurement of engagement and burnout: a two sample confirmatory factor analytic approach. J. Happiness Stud. 3, 71–92. doi: 10.1023/A:1015630930326

[ref51] Severiens S. E. Schmidt H. G. (2009). Academic and social integration and study progress in problem based learning. High. Educ. 58, 59–69. doi: 10.1007/s10734-008-9181-x

[ref52] van Alten D. C. Phielix C. Janssen J. Kester L. (2020a). Effects of self-regulated learning prompts in a flipped history classroom. Comput. Hum. Behav. 108:106318. doi: 10.1016/j.chb.2020.106318

[ref53] van Alten D. C. Phielix C. Janssen J. Kester L. (2020b). Self-regulated learning support in flipped learning videos enhances learning outcomes. Comput. Sci. Educ. 158:104000. doi: 10.1016/j.compedu.2020.104000

[ref54] van Gog T. Hoogerheide V. van Harsel M. (2020). The role of mental effort in fostering self-regulated learning with problem-solving tasks. Educ. Psychol. Rev. 32, 1055–1072. doi: 10.1007/s10648-020-09544-y

[ref55] Webster E. A. Hadwin A. F. (2015). Emotions and emotion regulation in undergraduate studying: examining students’ reports from a self-regulated learning perspective. Educ. Psychol. 35, 794–818. doi: 10.3846/bme.2015.297

[ref56] Wolters C. A. Taylor D. J. (2012). “A self-regulated learning perspective on student engagement,” in Handbook of Research on Student Engagement. eds. Christenson S. L. Reschly A. L. Wylie C. (New York: Springer), 635–651.

[ref57] Wong J. Baars M. Davis D. Van Der Zee T. Houben G. J. Paas F. (2019). Supporting self-regulated learning in online learning environments and MOOCs: a systematic review. Int. J. Hum. Comput. Interact. 35, 356–373. doi: 10.1080/10447318.2018.1543084

[ref58] Wyatt L. G. (2011). Nontraditional student engagement: increasing adult student success and retention. J. Contin. High. Educ. 59, 10–20. doi: 10.1080/07377363.2011.544977

[ref59] Xu Y. Guo P. Zhou W. Han J. (2019a). When does future work self predict work engagement: the boundary conditions of person-vocation fit and trust in supervisor. Int. J. Ment. Health Promot. 21, 31–44. doi: 10.32604/IJMHP.2019.010742

[ref60] Xu J. Xie B. Yang Y. Maharjan D. (2019b). Facilitating newcomers' work engagement: the role of organizational socialization and psychological capital. Int. J. Ment. Health Promot. 21, 69–80. doi: 10.32604/IJMHP.2019.010708

[ref61] Yeh Y. C. Kwok O. M. Chien H. Y. Sweany N. W. Baek E. McIntosh W. A. (2019). How college students’ achievement goal orientations predict their expected online learning outcome: the mediation roles of self-regulated learning strategies and supportive online learning behaviors. Online Learn. J. 23, 23–41. doi: 10.24059/olj.v23i4.2076

[ref62] Yukselturk E. Bulut S. (2007). Predictors for student success in an online course. J. Educ. Technol. Soc. 10, 71–83.

[ref63] Zhang Z. Cao T. Shu J. Liu H. (2020). Identifying key factors affecting college students’ adoption of the e-learning system in mandatory blended learning environments. Interact. Learn. Environ. 1–14. doi: 10.1080/10494820.2020.1723113

[ref64] Zhang H. Song W. Shen S. Huang R. (2014). The effects of blog-mediated peer feedback on learners’ motivation, collaboration, and course satisfaction in a second language writing course. Australas. J. Educ. Technol. 30, 670–685. doi: 10.14742/ajet.860

[ref68] Zhao C. Shi C. Zhang L. Zhai Z. Ren Z. Lin X.. (2020). Establishment of online platform for psychological assistance during a public health emergency. Int. J. Ment. Health Promot. 22, 123–132. doi: 10.32604/IJMHP.2020.011077

[ref65] Zheng B. Zhang Y. (2020). Self-regulated learning: the effect on medical student learning outcomes in a flipped classroom environment. BMC Med. Educ. 20:100. doi: 10.1186/s12909-020-02023-6, PMID: 32234040 PMC7110809

[ref66] Zimmerman B. J. (1986). Becoming a self-regulated learner: which are the key subprocesses? Contemp. Educ. Psychol. 11, 307–313. doi: 10.1016/0361-476X(86)90027-5

